# Disease-associated astrocytes and microglia markers are upregulated in mice fed high fat diet

**DOI:** 10.1038/s41598-023-39890-0

**Published:** 2023-08-09

**Authors:** Li Lin, Rashmita Basu, Debolina Chatterjee, Andrew T. Templin, Jonathan N. Flak, Travis S. Johnson

**Affiliations:** 1 Indiana Biosciences Research Institute, Indianapolis, IN USA; 2 Department of Pharmacology and Toxicology, Indiana University School of Medicine, Indianapolis, IN USA; 3 Department of Biostatistics and Health Data Science, Indiana University School of Medicine, Indianapolis, IN USA; 4 Division of Endocrinology, Department of Medicine, Richard L. Roudebush VA Medical Center, Indiana University School of Medicine, Indianapolis, IN USA

**Keywords:** Bioinformatics, Gene expression

## Abstract

High-fat diet (HFD) is associated with Alzheimer’s disease (AD) and type 2 diabetes risk, which share features such as insulin resistance and amylin deposition. We examined gene expression associated with astrocytes and microglia since dysfunction of these cell types is implicated in AD pathogenesis. We hypothesize gene expression changes in disease-associated astrocytes (DAA), disease-associated microglia and human Alzheimer’s microglia exist in diabetic and obese individuals before AD development. By analyzing bulk RNA-sequencing (RNA-seq) data generated from brains of mice fed HFD and humans with AD, 11 overlapping AD-associated differentially expressed genes were identified, including *Kcnj2*, *C4b* and *Ddr1*, which are upregulated in response to both HFD and AD. Analysis of single cell RNA-seq (scRNA-seq) data indicated *C4b* is astrocyte specific. Spatial transcriptomics (ST) revealed *C4b* colocalizes with *Gfad*, a known astrocyte marker, and the colocalization of *C4b* expressing cells with *Gad2* expressing cells, i.e., GABAergic neurons, in mouse brain. There also exists a positive correlation between *C4b* and *Gad2* expression in ST indicating a potential interaction between DAA and GABAergic neurons. These findings provide novel links between the pathogenesis of obesity, diabetes and AD and identify *C4b* as a potential early marker for AD in obese or diabetic individuals.

## Introduction

Alzheimer’s disease (AD) is the most common cause of dementia and is one of the leading causes of death in United States^1,2^. AD is characterized by gradual memory decline, the deposition of Aβ plaques, and the formation of neurofibrillary tangles which then lead to neuron loss and cognitive dysfunction^3,4^. Currently, there is no cure for AD, and its pathogenesis remains poorly understood. There are two broad categories of cells in the central nervous system (CNS): neurons and glial cells. Glial cells, which consist of astrocytes, microglia and oligodendrocytes, are the most abundant cells in the CNS^5^. Unlike neurons, glial cells are capable of mitosis, and have reduced blood–brain barrier and protective mechanisms.

Recent evidence suggests that glial cells play a pivotal role in the progression of neurodegenerative diseases^6,7^, and there is increasing interest in studying the contribution of astrocytes and microglia to the development and progression of AD. Astrocytes are key for maintaining brain homeostasis, supporting neurons, regulating synapses, neurotransmitter uptake and recycling, maintenance of blood–brain barrier (BBB) and cell signaling^8–14^. Microglia, CNS resident macrophages, can be activated by Aβ in AD pathogenesis, and once activated they can produce inflammatory mediators and may damage surrounding tissues^15,16^. Indeed, changes in microglia function contribute to brain aging as well as AD severity and progression^17^.

Recently, disease-associated astrocytes (DAA) and disease-associated microglia (DAM) have been identified in mouse models of AD. DAA appear in the early stage of AD before cognitive decline and increase as AD progresses^18^. In addition to Apolipoprotein E (*Apoe*), other genes associated with AD such as Clusterin (*Clu*) and FERM Domain Containing Kindlin 2 (*Fermt2*) are expressed in DAA clusters as well^19^. DAM contain many up-regulated genes associated with AD such as *Apoe*, Lipoprotein Lipase (*Lpl*) and Triggering Receptor Expressed On Myeloid Cells 2 (*Trem2*)*,* and they are primarily detected at sites of neurodegeneration in the brain^16,20^. Gene expression in human Alzheimer’s microglia (HAM) identified by Srinivasan et al. corresponds closely to that observed in DAM, with these both being significantly different from normal microglia^21^. HFD has damaging effects on the brain^22–24^. Studies in both human and rodents have shown that HFD intake is related with worse performance on cognitive tasks, possibly due to insulin resistance, oxidative stress, inflammation or reduced BBB integrity^25–29^. In mouse models of AD, HFD accelerates AD-like pathology and aggravates cognitive impairment, and mice on a long-term HFD have impaired memory and altered brain structure^22^. Neuroinflammation is a key component of AD progression^30^ and long-term HFD is known to promote inflammation in both the CNS and the periphery^23,24^. HFD can trigger proinflammatory functions of microglia^31^, and increased gliosis and markers of neuronal injury are observed in both obese humans and rodent HFD models^32,33^. However, the role of HFD in promoting DAA or DAM gene expression phenotypes has not yet been evaluated.

Currently, AD diagnosis is based on cognitive or behavioral assessment and laboratory tests including cerebrospinal fluid examination and brain imaging tests, and may also incorporate molecular biomarkers for enhancing the certainty of AD^34^. RNA-seq is a powerful tool which can uncover the molecular mechanisms and gene functions, aiding in the discovery of potential biomarkers for AD and providing additional targets for early intervention. We hypothesize that some of the gene expression changes that occur in AD-associated microglia and astrocytes are present during diabetes and obesity, and that these changes in gene expression contribute to cognitive decline and AD. The preoptic area of the brain has received extensive attention in the study of obesity with respect to regulation of food intake, response to temperature change and coordinated regulation of sleep^35,36^. Sleep–wake disturbances are prevalent among individuals with AD, suggesting a potential correlation between preoptic areas and AD^37^. A reduction of cell number has been reported in the ventrolateral preoptic/intermediate nucleus in older adults, which has been linked to AD as well^36^. In addition, the nearby bed nucleus of the stria terminalis (dorsal to the ventrolateral preoptic area) receives less galanin-containing fibers in aging and AD patients^39^. Despite its potential role in AD, the preoptic area is understudied in this context. Therefore, this study will identify glial cell targets for further investigation of the mechanisms underlying preoptic area dysfunction in AD.

## Methods

### Analysis overview

**Figure 1:**
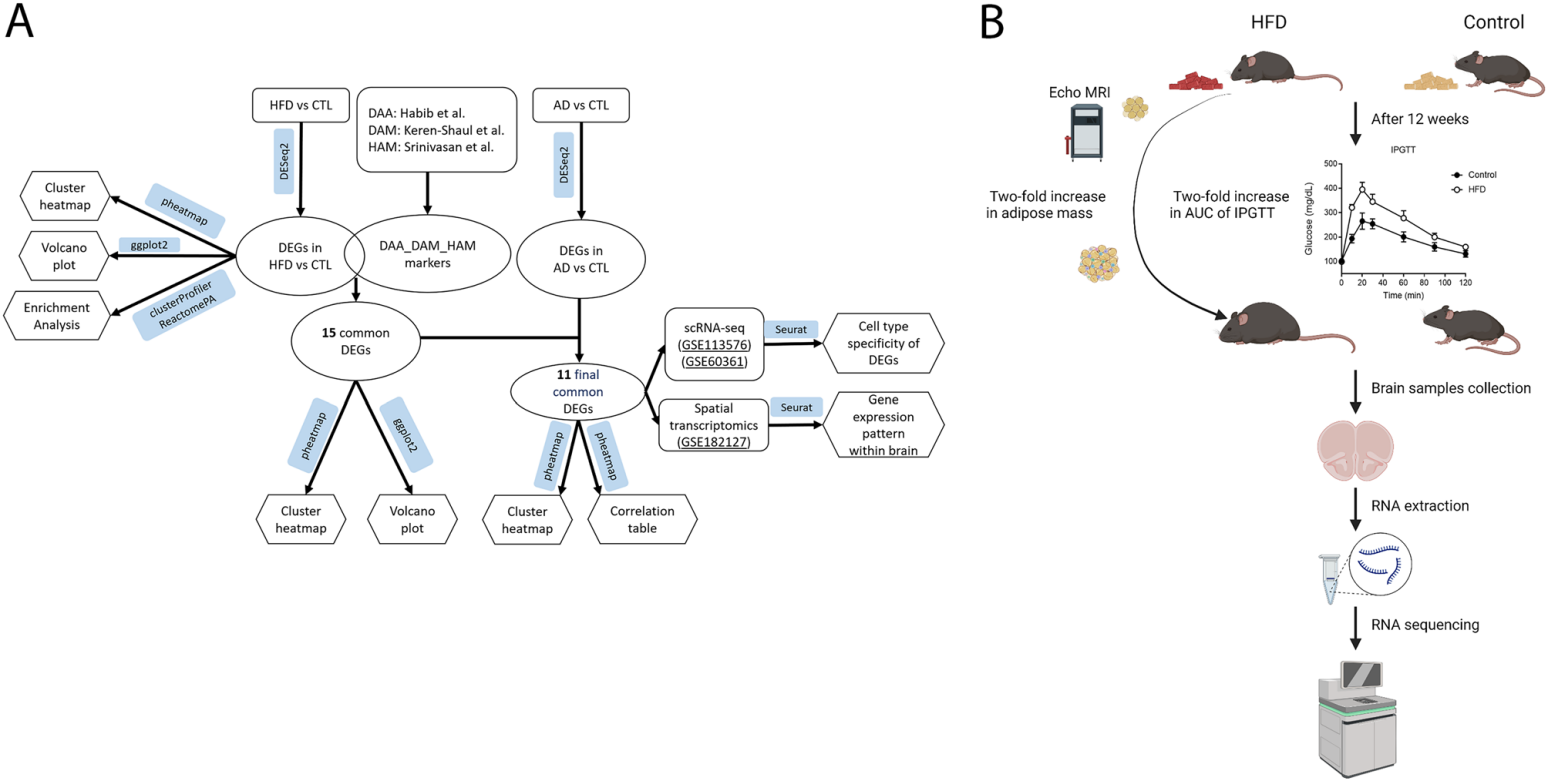
Experimental workflow. (**A**) The workflow of RNA-seq analysis. (**B**) The procedures of RNA-seq data generation from the mouse model of HFD-induced obesity and diabetes.

To interrogate the potential interactions between the pathogenesis of obesity, diabetes and AD, this study utilized the following datasets: (1) bulk RNA-seq datasets from our HFD-fed mouse brains (n = 8) and AD human brains (n = 682) obtained from the Mount Sinai Brain Bank (MSBB), (2) previously identified DAA, DAM and HAM markers, and (3) publicly available scRNA-seq datasets (n = 6) (GSE113576), (n = 67) (GSE60361) and spatial transcriptomics (ST) dataset (n = 7) (GSE182127). All data analysis was performed in R. We utilized *DESeq2* package to identify differentially expressed genes (DEGs)^40^. The *pheatmap* package was employed to generate heatmaps^41^. Volcano plots were made using *ggplot2* package to visualize the expression patterns of these DEGs^42^. We performed enrichment analysis using the *clusterProfiler* and *ReactomePA* packages to identify enriched biological pathways and functions associated with DEGs^43,44^. DAA, DAM and HAM markers found in previous studies were used in our bulk RNA-seq analysis^16,18,21^. They were employed to identify the common genes among DEGs found in HFD-fed mice. Subsequently, these shared genes were then used to screen the overlapping DEGs identified in MSBB bulk RNA-seq dataset from AD-patients’ brains. To investigate shared upregulated genes in response to both HFD and AD, we analyzed publicly available scRNA-seq and ST datasets to study cell type specificity and gene expression patterns in the preotic brain regions (Fig. 1A).

### Animals

We generated RNA-seq data from a mouse model of HFD-induced obesity and diabetes (Fig. 1B). The procedures were approved by the Institutional Animal Care and Use Committee at the Indiana University School of Medicine (Indianapolis, IN). All methods were performed in accordance with the relevant guidelines and regulations. All mice were provided with chow and water ad libitum, unless noted otherwise, and kept in a temperature-controlled (22 °C) room on a 12-h light–dark cycle. 7–9 weeks old C57BL6/J male mice were acquired from Jackson laboratory (Bar Harbor, ME). After a week of recovery, each cage of five mice were placed on either control diet (D12450K, 10 kcal% fat, 70 kcal% carbohydrate, 20% protein) or HFD (D12451, 45 kcal% fat, 35 kcal% carbohydrate, 20% protein) from Research Diets (New Brunswick, NJ). After 12 weeks, mice were tested for body fat composition on Echo MRI. The next week, mice were tested for intraperitoneal glucose intolerance (IPGTT; 2 g/kg body weight). Only mice with a two-fold increase in adipose mass and area under the curve (AUC) of IPGTT were included. To collect tissue, mice were deeply anesthetized using isoflurane, and brain removed. 1 mm sections were dissected with fresh razor blades using a 1 mm mouse brain matrix (Braintree Scientific). Sections were dissected based on the Allen brain atlas, then placed in a tube on dry ice. Four dissected preoptic regions were removed and split in half to separate the dorsal and ventral preoptic regions, due to the distinct differences in functions between the two: dorsal can induce behaviors, while ventral controls the functions for peripheral organs. When ready, RNA was extracted using Trizol Plus RNA Purification (Invitrogen) with column DNAse 1 treatment (Zymo Research) and then sent for RNA-seq by the Indiana University School of Medicine Center for Medical Genomics. Samples for RNA-seq data came from 5 mice on HFD and 3 mice on control diet. We collected one dorsal preoptic region sample and one ventral preoptic region sample from each mouse. Each sample underwent an RNA Integrity Number (RIN) assessment, and only the samples with a RIN value above 6.5 were selected for library preparation. KAPA RNA HyperPrep Kits (KK8581, Roche) were used to prepare RNA libraries and Illumina NovaSeq 6000 sequencer was used to perform sequencing.

### DEGs identified from HFD mice and functional enrichment analysis

The R package *DESeq2* was used to identify DEGs. Firstly, the count data and meta data were loaded into R, and a DESeq dataset object was constructed using *DESeqDataSetFromMatrix* function, followed by the *DESeq* function to perform the analysis. To filter out low count genes and address the issue of unreliably large log fold changes, we applied the *lfcShrink* function using the apeglm implementation^38^. The thresholds of DEGs were set at a false discovery rate (FDR)-adjusted *p*-value < 0.05 and absolute log2 fold change (log_2_FC) > 0.3. Heatmaps and volcano plots were generated using *pheatmap* and *ggplot2*, respectively, to visualize the hierarchical clustering of DEGs in the dorsal and ventral preoptic regions. When using *pheatmap* function to create the cluster heatmap for the dorsal preoptic region samples, screening criteria of adjusted *p*-value < 0.05 and |log_2_ FC|> 0.63 were applied and the numeric matrix of the values was converted to log2 scale to improve visualization. Functional enrichment analysis was separately performed for the dorsal and ventral preoptic regions using gene ontology (GO) molecular function (MF), GO biological process (BP), GO cellular component (CC), Kyoto encyclopedia of Genes and Genomes (KEGG) and Reactome pathway (REAC) analyses. To conduct the enrichment analysis, we use the *enrichGO* and *enrichKEGG* functions in *clusterProfiler* package and *enrichPathway* function in *ReactomePA* package. For all enrichment analyses, Benjamini–Hochberg adjusted FDR was used to correct the *p*-values and considered an FDR cutoff of 0.05 as significant for enriched terms.

### Clinical features and DEGs identified from MSBB dataset

We obtained clinical features and RNA-seq data from control and AD brains samples in MSBB AD study^45^. There were 273 controls (136 females and 137 males) and 409 cases (265 females and 144 males) with a definitive AD diagnosis. The majority of samples, 547 were from individuals of White race, while 76 were Black, 52 were Hispanic, 3 were Asian, and 4 were unknown. For our study, we also considered several clinical phenotypes, including clinical dementia rating (CDR), age of death (AOD), mean plaque density (PlaqueMean) and Braak AD-staging score (bbscore). The CDR ranged from 0 to 5 with higher values indicating more severe cognitive impairment. The average CDR for control and AD samples were 1.0 and 3.1 respectively. The AOD of all samples ranged from 61 to 90 with a mean of 82.5 ± 7.9. Mean plaque density reveals the severity of Aβ deposition^46^, with a mean 0.7 ± 1.6 for controls and 15.3 ± 9.0 for AD samples. The bbscore is associated with neurofibrillary tangles and its range is from 0 to 6, with a mean 1.9 ± 1.0 for controls and 5.2 ± 1.2 for AD samples. To identify DEGs between AD cases and controls, we applied an FDR-adjusted *p*-value < 0.05 and absolute log_2_ FC > 0.18 as the significance thresholds.

### Identification and correlation analysis of overlapping DEGs in HFD mice and human AD studies

Initially, we identified DEGs in HFD mice and compared them to the lists of DAA, DAM and HAM markers (Supplementary Table S1) acquired from previous research^16,18,21^. By finding the intersection, we obtained a set of 15 DEGs. They were further compared with the DEGs identified from bulk RNA-seq data obtained from MSBB, resulting in 11 overlapping DEGs (Table 1). To investigate the potential correlation between these 11 overlapping DEGs and AD clinical phenotypes, we performed correlation analysis. Clinical phenotypes include CDR, AOD, PlaqueMean, and bbscore.

**Table 1:** Cell type-specificity of DEGs in HFD and AD study.

Gene	AD-associated cell type	HFD versus control	AD versus control	Specificity to major cell type
*Gdpd2	DAA	Downregulated	Upregulated	Astrocyte specific
*C4b	DAA	Upregulated	Upregulated	Astrocyte specific
*Sbno2*	DAA	Downregulated	Upregulated	Astrocyte nonspecific
*Col16a1*	DAA	Downregulated	Upregulated	Astrocyte nonspecific
*Ddr1*	DAA	Upregulated	Upregulated	Astrocyte nonspecific
*Adam8	HAM	Downregulated	Upregulated	Microglia specific
*Csf2ra	DAM	Downregulated	Upregulated	Microglia specific
*Dnajb14*	DAM	Upregulated	Downregulated	Microglia nonspecific
*Gas7*	DAM	Upregulated	Downregulated	Microglia nonspecific
*Lgi2*	DAM	Upregulated	Downregulated	Microglia nonspecific
*Kcnj2*	DAM	Upregulated	Upregulated	Microglia nonspecific

*Gdpd2, C4b, Adam8 and Csf2ra* have been marked with an asterisk (*) considered cell type specific.

### Cell type-specificity of DEGs using scRNA-seq

A publicly available dataset (GSE113576) of scRNA-seq was retrieved as reference to generate gene expression signature, which then was used to infer if genes of interest are cell type-specific to astrocytes or microglia^47^. The dataset was generated from the hypothalamic preoptic region in healthy male and female mice. The *Seurat* package in R was used to analyze scRNA-seq dataset and performed quality control, data normalization, feature selection, dimensionality reduction and cell clustering^48–51^. Filtering was applied in order to remove low-quality cells by excluding cells expressing less than 200 or greater than 2,000 genes as well as cells with greater than 5% mitochondrial gene expression. The remaining cells were normalized by using “LogNormalize” method and multiplied by 10,000. The top 2,000 highly variable genes were then identified using *FindVariableFeature* function, and a linear transformation was applied to center and scale data matrix using *ScaleData* function. Principle component analysis was performed, and an elbow plot was used to select the first ten principal components for t-distributed stochastic neighbor embedding analysis and clustering. The resulting 14 clusters were then annotated based on cell type information provided in “Cell.class” column of the metadata file. The proportion of each cell type in the *Seurat* object was calculated by dividing the frequency table that showed the number of cells in each cell type by the total number of cells. We then calculated the counts per million for the single cell dataset to evaluate the mean expression of genes of interest in each cell type. A t-test was performed to compare the expression of the gene in a specific cell type with the expression in all other cell types combined. A *p*-value less than 0.05 is considered statistically significant.

### Ethics approval and informed consent

The study is reported in accordance with ARRIVE guidelines. No human subjects were recruited for this study. The animal experiments in this study were approved by the Committee on the Use and Care of Animals at the Indiana University School of Medicine (Indianapolis, IN).

## Results

### DEGs in HFD cohort

**Figure 2:**
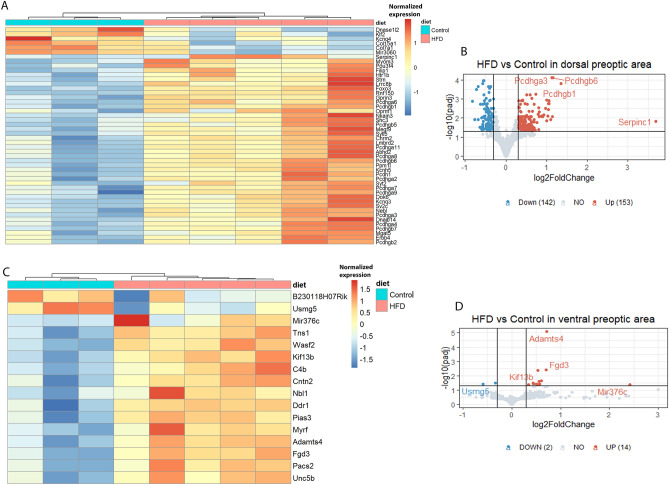
Heatmaps and volcano plots of up- and down- regulated genes in brains from mice fed HFD versus a regular diet. (**A**) DEGs identified in dorsal preoptic brain region are displayed in a heatmap (FDR-adjusted *p*-value < 0.05 and |log2 FC|> 0.63). (**B**) DEGs identified in dorsal preoptic brain region are displayed in a volcano plot (FDR-adjusted *p*-value < 0.05 and |log2 FC|> 0.3). A heatmap (**C**) and a volcano plot (**D**) show DEGs identified in the ventral preoptic region brain region (FDR adjusted *p*-value < 0.05 and |log2 FC|> 0.3). Note that in the heatmaps the control samples are in the left three columns, while the right five columns represent samples from mice on HFD. The color scales in heatmps (**A**) (**C**) represent normalized expression levels with performing *pheatmap*. In the heatmaps and volcano plots, up-regulated genes are shown in red, while down-regulated genes are shown in blue.

There were 311 DEGs identified from the HFD mice generated by us, with 295 DEGs obtained from the dorsal preoptic region (153 upregulated and 142 downregulated) (Fig. 2A, B). In contrast, only 16 DEGs (14 upregulated and 2 downregulated) are identified in the ventral preoptic region (Fig. 2C, D).

### Functional enrichment analysis of DEGs in HFD cohort

False positive DEGs related to microRNA were removed before functional enrichment analysis, which include *Mir3060* and *Mirg* from DEGs in dorsal preoptic area and *Mir376c* from DEGs in ventral preoptic area. GO term results for DEGs identified in dorsal preoptic region results are shown in Supplementary Table S2. GO analysis of CC categories suggested the majority of DEGs from the dorsal preoptic region are in the plasma membrane, cell periphery, cell membrane, synapse, neuron projection, collagen trimer and extracellular matrix. The main MF terms are extracellular matrix structural constituent, ion channel activity and transmembrane transporter activity. For BP, DEGs are enriched for cell–cell adhesion, extracellular structure organization, synaptic signaling, ion transport and stress response to acid chemicals. KEGG pathway analysis reveals that DEGs are enriched for protein digestion and absorption, ECM-receptor interaction pathways, focal adhesion, human papillomavirus infection and PI3K-Akt signaling pathway (Supplementary Table S3). The most enriched Reactome pathways include the formation and degradation of collagen and extracellular matrix, potassium channels, NCAM1 interactions, neuronal system, signal transduction and signaling by PDGF (Supplementary Table S4). Due to the limited number of DEGs identified from the ventral preoptic region, only GO term results for BP and CC are obtained, which are significantly enriched in central nervous system myelination, axon ensheathment in central nervous system and oligodendrocyte development (Supplementary Table S5).

### Overlapping DEGs in AD cohort and correlation with clinical phenotypes

**Figure 3:**
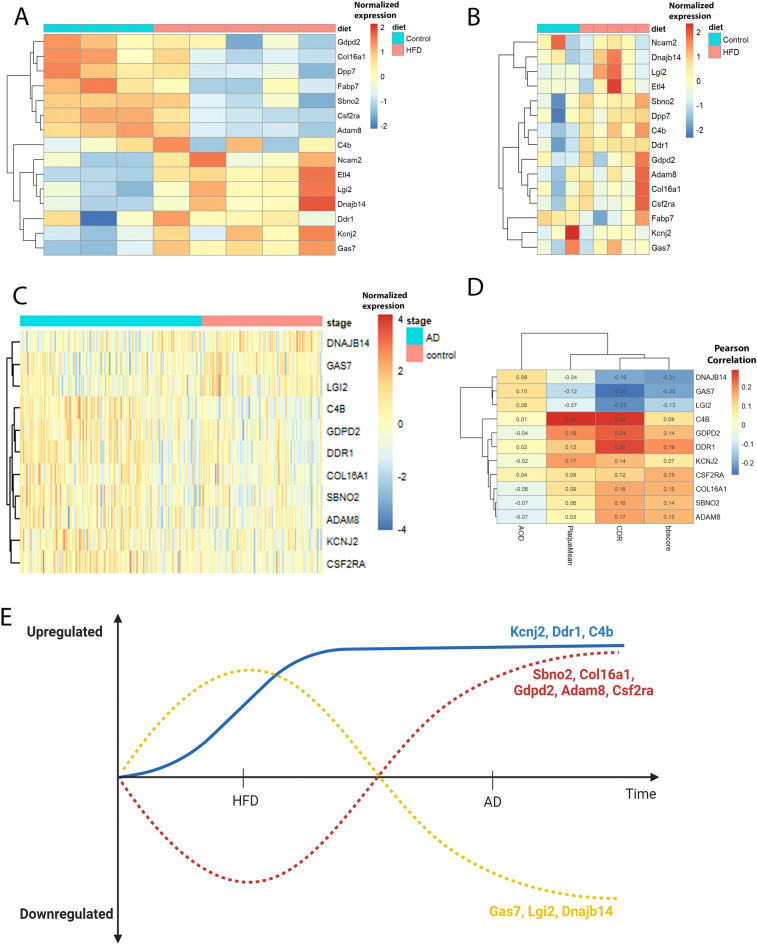
Hierarchical clustering heatmaps, correlation heatmaps and theoretical regulation of gene expression in the preoptic region. (**A**, **B**) Heatmaps showing the expression profile of 15 DEGs identified from HFD cohort in dorsal preoptic region (**A**) and ventral preoptic region (**B**) overlapping with DAA, DAM and HAM markers. The left 3 columns represent 3 control samples and the rest columns on the right are samples from HFD. (**C**) A heatmap for 11 common DEGs found among DAA, DAM and HAM markers, HFD and AD brain samples. The red and the blue color represent up-regulated or down-regulated genes respectively. The color scales in heatmaps (**A**) (**B**) (**C**) represent normalized expression levels with performing *pheatmap*. (**D**) Correlation heatmap using Pearson method between DEGs and the clinical phenotypes with Pearson’s correlation coefficient in each cell between DEGs and AD clinical phenotypes. The color scale displays Pearson correlation. Red color indicates positive correlation while blue color shows negative correlation. The darker the color means the stronger correlation. (**E**) The plots illustrate the theoretical changes in genes from consumption of high-fat diet to the development of AD.

We identified 15 DEGs out of 311 DEGs overlapping between HFD cohort and DAA, DAM or HAM markers. These 15 genes include *Ncam2, C4b, Ddr1, Sbno2, Fabp7, Col16a1, Gdpd2, Lgi2, Etl4, Kcnj2, Gas7, Dnajb14, Csf2ra, Dpp7, Adam8*. Their expression patterns were shown in heatmaps in the dorsal and ventral preoptic regions respectively (Fig. 3A, B). *C4b* and *Ddr1* were identified in the ventral preoptic region while others were identified in the dorsal preoptic region of HFD-fed mice brain. In the AD cohort, 7444 DEGs were identified from MSBB RNA-seq data with 4554 upregulated and 2890 downregulated. The heatmap and volcano plots were in additional file (Additional file 1, Supplementary Fig.S1). Due to the large number of samples and genes from AD human dataset (n = 682), we increased the significance threshold of log2FC from 0.18 to 0.8 for the heatmap to enhance visualization (FDR-adjusted *p*-value < 0.05 and absolute log2 FC > 0.80). We identified 11 DEGs by intersecting the 15 previously identified DEGs from HFD cohort with DEGs from AD cohort and they were *Dnajb14, Gas7, Lgi2, C4b, Gdpd2, Ddr1, Col16a1, Sbno2, Adam8, Kcnj2, Csf2ra* (Fig. 3C). These 11 DEGs also showed correlations with clinical features of AD (Fig. 3D). Clinical phenotypes, except AOD, showed statistically significant correlations with these DEGs with *p*-value < 0.01. Specifically, *C4b, Ddr1, Sbno2, Col16a1, Gdpd2, Kcnj2, Csf2ra, Adam8* were positively related to PlaqueMean, CDR and bbscore, while *Dnajb14, Gas7* and *Lgi2* exhibited negative correlation with the same variables. Our results and accumulating evidence show that obese and diabetic individuals are predisposed to AD^52–54^. We theorized individuals with diabetes or obesity may be at a potentially higher risk of developing AD later in life (Fig. 3E). It is plausible that the dysregulation of these genes over time in diabetic and obese individuals could potentially contribute to the development of AD. Further research is needed to fully understand the complex relationship between these factors.

### Identification of cell type-specific gene expression signature in the final 11 DEGs via scRNA-seq analysis

After conducting scRNA-seq analysis, we identified 14 types of cell clusters which consisted of 4.5% astrocytes, 1.5% endothelial cells, 0.3% ependymal cells, 3.6% excitatory neurons, 33.3% inhibitory neurons, 0.3% fibroblasts, 0.8% newly formed oligodendrocytes, 4.6% immature oligodendrocytes, 33.3% mature oligodendrocytes, 0.2% macrophages, 2.6% microglia, 2.8% mural cells, 1.1% ambiguous cells and 0.2% unstable cells. According to the average expression of these 11 genes in each cell type and *p*-value of t-test (Supplementary Table S6), we inferred whether DEGs were cell type-specific to astrocytes or microglia. In Table 1, we showed how cell type-specific gene expression changed in response to HFD or AD. *Kcnj2*, *Ddr1* and *C4b* were the only genes upregulated in both HFD and AD cohort study. Based on previous research, *Kcnj2* was considered as a DAM marker and *Ddr1* and *C4b* were taken as DAA markers. Our findings indicate *C4b* is astrocyte specific, while *Kcnj2* and *Ddr1* are not specific to microglia or astrocytes. The average expression of *Kcnj2* in microglia is low and *p*-value of its t-test is close to 1, which means the expression of *Kcnj2* in microglia is not significantly different from other cells. *Ddr1* is highly and significantly expressed in astrocytes with a *p*-value less than 0.01. However, the expression of *Ddr1* in oligodendrocytes is three times higher than its expression astrocytes. Hence, *Ddr1* is considered non-specific to astrocytes though it is still a promising target. *C4b* shows the moderately higher mean expression in ependymal cells and macrophages compared to astrocytes, but they account for very low percentages of the total cells (0.3% ependymal cells and 0.2% macrophages), indicating that the majority of expression is likely not from either ependymal cells or macrophages. The expression of *C4b* is more likely coming from astrocytes due to its high average expression in astrocytes which constitute 4.5% of the total cells. Additionally, the t-test results suggest that the expression of *C4b* is significantly greater in astrocytes than other cell types, with a *p*-value less than 0.01. It is likely that *C4b* is a reliable astrocyte marker. For genes that show differential regulation in the HFD and AD cohort in opposite directions, it is highly suggestive that *Gdpd2* is an astrocyte marker, as it has the highest mean expression in this cell type and a significant *p*-value of less than 0.01. Similarly, *Adam8* and *Csf2ra* appear to be highly expressed in microglia, as indicated by their high mean expression in this cell type and significant *p*-value of less than 0.01. While macrophages also exhibit high expression of *Adam8* and *Csf2ra*, they represent a very small proportion of the total cell population in this dataset, supporting *Adam8* and *Csf2ra* are more likely to serve as microglia markers.

Additional validation of the cell type-specificity for *C4b*, *Gdpd2*, *Adam8* and *Csf2ra* was conducted in public dataset GSE60361 (scRNA-seq of mouse cerebral cortex) by applying the same method described in Sect.  2.7 (Table 2)^55^. Both *C4b* and *Gdpd2* were expressed the most in astrocytes with *p*-values less than 0.01, and *Adam8* and *Csf2ra* displayed the highest mean expression in microglia (Supplementary Table S7). Our findings align with a previous study by Cahoy et al., which demonstrated significant enrichment of *C4b* and *Gdpd2* in astrocytes^56^.

**Table 2:** Cell type-specificity validation of *C4b*, *Gdpd2*, *Adam8* and *Csf2ra* in public datasets.

Gene	GSE113576		GSE60361	
	Astrocyte specific	Microglia specific	Astrocyte specific	Microglia specific
*C4b*	✓		✓	
*Gdpd2*	✓		✓	
*Adam8*		✓		✓*
*Csf2ra*		✓		✓*

*High mean expression and not significant at (*p* < 0.05).

### Spatial gene and protein expression of DAA markers

From the previous analysis we identified *C4b* and *Ddr1* as our highest confidence markers for progression from diabetic state to AD. We then determined whether these genes and proteins were expressed in our tissue regions using the human protein atlas. The details for the methods used in the ST analysis and supplementary figures are provided in additional file (Additional File 1). We found that *C4b*, *Ddr1, Gad2,* and *Gfap* protein can be expressed in the preoptic area (Supplementary Fig. S2A-D). This supports the presence of both GABAerigic neurons and astrocytes in the preoptic area^47^, which is in line with what we observe in our ongoing studies into the region. There is not currently data available for ST from the preoptic area. The closest region with such data is the hypothalamus. We used the newly available 10X Genomics Xenium (10X Genomics datasets) and Visium data (10X Genomics datasets; GSE182127) from coronal sections of mouse brains to study the colocalization of astrocyte (*Gfap*), DAA (*C4b*), GABAergic neuron (*Gad1* and *Gad2*), and Glutamatergic neuron (*Slc17a7* and *Slc17a6*) markers in coronal sections of mouse brains that intersect the hypothalamus. We found that in the single cell resolution Xenium data, the astrocyte marker *Gfap* frequently colocalize with the GABAergic neuron marker *Gad2* (Supplementary Fig. S2E-F). Furthermore, in the 10 × Genomics Visium data, we found that *Gfap, C4b*, and *Gad2* frequently colocalize and postulate this may indicate that astrocytes with a DAA-like phenotype colocalize with GABAergic neurons in the hypothalamus (Supplementary Fig. S3-9). We recognize the limitation of not having this data from the preoptic area but believe that this data still adds context to the likelihood of an interaction between DAA-like cells and GABAergic neurons in the mouse brain. More single cell resolution spatial transcriptomic data from the preoptic area would be ideal for the study of these interactions as it becomes available in the future.

## Discussion

This study investigated the transcriptomic changes of AD-associated astrocytes and microglia in the brains of mice fed HFD and humans with AD. The mouse brain samples were obtained from dorsal and ventral preoptic regions, which are known to have distinct functions. The dorsal preoptic region is enriched with the bed nucleus of the stria terminalis, which is thought to modulate autonomic, neuroendocrine and behavioral responses, including cardiovascular and hypothalamus–pituitary–adrenal axis activity^57^. The ventral preoptic region, on the other hand, is involved in regulating core body temperature, sleep, food intake, and is therefore related to body weight homeostasis^35^. We identified 295 DEGs in response to HFD feeding in the dorsal preoptic region, while only 16 DEGs were found in the ventral preoptic region. This suggests that the dorsal preoptic region may play a more important role than the ventral preoptic region in regulating HFD-related gene expression. DAA, DAM, or HAM markers that were both differentially expressed in response to HFD and in brains of individuals with AD were examined in this study. As a result, 11 DEGs were identified. Interestingly, only three of them were regulated in the same direction in response to both HFD and AD, which are complement 4B (*C4b*), potassium inwardly rectifying channel subfamily J member 2 (*Kcnj2*) and discoidin domain receptor1 (*Ddr1*). In both HFD and AD studies, they were upregulated, and they were positively related to measures of AD severity CDR, PlaqueMean and bbscore. It is possible that these genes are regulated by HFD and they mediate the progression to AD in individuals predisposed to the disease.

The exact cellular interactions mediating HFD and diabetic predisposition for AD are still poorly understood. However, GABAergic interneurons dysfunction has been implicated in the pathogenesis of AD^58^. Our findings from ST data analysis revealed frequent colocalization of astrocytes (*Gfap*), *C4b* and GABAergic neurons (*Gad2*) in the hypothalamus, indicating the possible interaction between astrocytes with an AD-associated phenotype and GABAergic neurons in this brain region. It has also been reported that there is communication between GABAergic interneurons and astrocytes^59^. Astrocytes expressing *C4b* in AD may be associated with the dysfunction of GABAergic neurons and contribute to the development of AD. In the CNS, the complement system is a part of innate immunity known to protect against neuroinflammation and to be essential for brain development^60^. It contributes to the clearance of pathogens to limit inflammation^61^. However, dysregulation of the complement cascade can lead to neurodegenerative diseases^62^. While the complement cascade can have both inflammatory and neuroprotective functions, complement activity has been associated with the loss of synapses in mouse AD models^63^. *C4b* together with *C4a* encode the complement protein (C4). There is evidence to suggest that mice without C4 have impaired synaptic pruning, and an increase in C4 may contribute to schizophrenia risk due to excessively pruning synapses during the development of brain^64^. It may increase the risk of AD as well. In AD patients, the copy number of *C4a* and *C4b* is significantly increased compared to healthy controls, which may explain the high *C4* protein expression observed in AD patients^65^. Upregulation of *C4b* has been observed in aged astrocytes, suggesting its involvement in the alterations of astrocytes induced by aging that potentially contribute to cognitive decline^66,67^. And study has found a variant of the *C4b* gene, *C4b2* is associated with an increased risk of developing AD^68^. In addition, there is evidence showing the complement system, including *C4b*, may contribute to the development of AD through inflammation and damage to brain cells. Complement system can be activated in the brain in response to Aβ deposition, and this activation can lead to inflammation and damage to neurons^69^. Furthermore, studies in animal models have suggested that blocking the complement system may reduce the accumulation of Aβ and improve cognitive function^70^. Our data are among the first to identify *C4b* as being upregulated in astrocytes in response to HFD feeding, suggesting HFD-induced *C4b* may contribute to AD pathogenesis.

*Kcnj2* is a gene that encodes a lipid-gated ion channel, which enables potassium ions to enter cells. This function is consistent with potassium ion transmembrane transport function revealed by our GO and Reactome pathway enrichment analysis in DEGs identified from our mouse HFD study. While *Kcnj2* has not been directly linked to AD, there is some evidence to suggest ion channel dysfunction, including dysfunction of potassium channels like *Kcnj2* may be involved in the development and progression of AD. Studies have shown that ion channel function is altered in the brains of individuals with AD, which can affect the normal functioning of neurons and other cells in the brain^71^. In particular, alterations in potassium channel function have been implicated in neuronal hyperexcitability, which can contribute to the degeneration of neurons in the brain^72^. Previous research has shown that potassium channel is important in regulating microglia functions in neurodegenerative diseases^73^. It has been reported that microglia express various types of K^+^ channels including *Kcnj2*^74^. *Kcnj2* channel activity is essential for Ca^2+^ signaling in microglia, and it is necessary for the proliferation and migration of these cells^75^. *Kcnj2* is also expressed in sour-sensing taste cells^76^. It is known that HFD is related to the increased risk of AD. Taste plays a crucial role in food choice, and interestingly, gustatory sensitivity decreases as AD progresses, including an increase in the sour threshold^77^. Elderly individuals who carry *Kcnj2-rs236514* have a higher tendency to prefer sour tastes and have more severe cognitive impairment^76^. Our data indicate that HFD leads to upregulation of *Kcnj2* in astrocytes and suggests HFD-induced *Kcnj2* expression may play a role in the development of AD.

Enriched pathways involved in collagen degradation or formation were identified through Reactome analysis. *Ddr1* is one of two members of *Ddr* family of tyrosine kinase receptors that participates in pathways regulating cellular processes such as cell growth, differentiation and metabolism^78^. *Ddr1* may act as a collagen receptor and can be activated by various types of collagen^79^. The expression of *Ddr1* is enriched in CNS. In addition to astrocytes, it also expresses among myelinating oligodendrocytes, myelin and neurons^80^. *Ddr1* is important for the regulation of microglial activity^81^. It has been reported that *Ddr1* mediates inflammatory response of microglia induced by collagen^82^, which may contribute to the neuroinflammation and neurodegeneration. Upregulation of DDRs, including *Ddr1*, has been observed in post-mortem brains of individuals with AD and Parkinson’s diseases (PD)^83^, and previous studies show that the inhibition or deletion of *Ddr1* can lower toxic proteins such as α-synuclein, tau, and Aβ in AD and PD models, promoting autophagy both in vivo and vitro^83,84^. Our data indicates that HFD increases the expression of *Ddr1* in astrocytes and microglia and suggests that the upregulation of *Ddr1* caused by HFD may contribute to the pathogenesis of AD.

Except for *C4b, Kcnj2* and *Ddr1*, the other DEGs identified in our study were regulated in opposite directions in HFD versus AD cohorts. Growth arrest specific protein 7 (*Gas7*) is upregulated in HFD cohort while it is downregulated in AD cohort. *Gas7* is reported to be expressed in neuronal cells and be involved in neuronal development and neurotransmitter release^85^. Through binding tau to alter its conformation, *Gas7* inhibits the formation of fibrils^86^. It is reported that the level of *Gas7* decreased in the brains from patients with AD^87^. It indicates *Gas7* may protect from AD which is consistent with our findings that the downregulation of *Gas7* in AD cohort and its strongly positive correlation with greater AOD and negatively link with CDR and bbscore. However, elevated level of *Gas7* has also been implicated in pathogenesis of AD progression, as it may interfere with kinesin motility on microtubules and disrupt the homeostasis of tau in CNS^88^.

Previous work has shown the Adams family of zinc metalloproteinases, including *Adam8*, play a role in the positive side of proteolysis, as it enhances the degradation of Aβ and decreases its generation in AD^89^. Strawberry notch homolog 2 (*Sbno2*) is an inflammatory response gene. In genome-wide association study (GWAS) studies, it has been identified in AD-risk loci^90^. Furthermore, *Sbno2* is also found to be associated with BMI and body weight^91^. Colony stimulating factor 2 receptor subunit alpha (*Csf2ra*) is likely to be a reliable DAM marker in our study, and the role of *Csf2ra* as a DAM marker has also been mentioned in another research^92^. *Csf2ra* mediates the function of granulocytes and macrophages and its protein expression is reduced in human AD brains^92^. *Col16a1* is a member of the FACIT collagen family and serves to mediate cell attachment and maintains the integrity of the extracellular matrix. It is also expressed in perivascular astrocytes, which regulate blood–brain barrier physiology^93^. Our study results displayed the downregulation of *Col16a*, which corresponds to a research in frontotemporal dementia and amyotrophic lateral sclerosis^94^. It is demonstrated in our research that *Gdpd2* can be a reliable DAA marker, and it is also expressed in oligodendrocyte precursor cells. Moreover, It negatively regulates the proliferation of oligodendrocyte precursor cells through the release of ciliary neurotrophic factor receptor α^95^.

Sex hormones and adipokines play importance roles in modulating insulin sensitivity and glucose metabolism^96^. Due to the absence of a potential protective effect of estrogen and higher amount of visceral and hepatic adipose tissue, men tend to have higher levels of insulin resistance compared to women^96^. We acknowledge that our study has a limitation in that it focused solely on male mice in the HFD study. We recognize the importance of studying both male and female subjects to obtain a more comprehensive understanding of the topic. Future investigations should consider including female mice to provide a more complete analysis. In summary, we investigated the dysregulation of DAA, DAM and HAM markers in response to HFD and AD, and we examined the relationship between obesity, diabetes and AD. We identified shared genes, particularly *C4b*, which may shed light on the underlying mechanism in the preoptic region for the development of AD and highlight the need for more scRNA-seq and ST data to be generated from underreported brain regions like the preoptic area. Our findings emphasize the importance of studying these shared genes for a better understanding of the link between metabolic disorders and AD.

### Supplementary Information

Supplementary Information 1.

Supplementary Table S1.

Supplementary Table S2.

Supplementary Table S3.

Supplementary Table S4.

Supplementary Table S5.

Supplementary Table S6.

Supplementary Table S7.

## Data Availability

The mouse RNA-seq data can be accessed at Synapse (https://www.synapse.org/#!Synapse:syn51929806). Data can be found at the following links: Fastq files: https://www.synapse.org/#!Synapse:syn52047798. RNA-seq count data: https://www.synapse.org/#!Synapse:syn51929900. Metadata: https://www.synapse.org/#!Synapse:syn52050369. A data use agreement is required to access the data and the agreement can be obtained from SAGE Bionetworks, who created and manage Synapse.
