# Supplementary material for: Disease-associated astrocytes and microglia markers are upregulated in mice fed high fat diet
Source: Sci Rep. 2023 Aug 9;13:12919. doi: 10.1038/s41598-023-39890-0 (PMC10412627; doi:10.1038/s41598-023-39890-0)
Supplement: Supplementary file 1 — Supplementary Information 1. [file 41598_2023_39890_MOESM1_ESM.docx]

**Supplementary Materials for “Disease Associated Astrocytes and Microglia Markers are Upregulated in Mice Fed High-Fat Diet”**

Li Lin, Rashmita Basu, Debolina Chatterjee, Andrew T. Templin, Jonathan N. Flak, Travis S. Johnson

Corresponding author: Travis S. Johnson

Email: [johnstrs@iu.edu](mailto:johnstrs@iu.edu)

Supplementary Method: 1 section

Supplementary Figures: 9 figures

**Supplementary method**

*Spatial transcriptomics analysis*

We have the acquired the Visium ST datasets of the Mouse brain coronal section from GEO (url = <https://www.ncbi.nlm.nih.gov/geo/query/acc.cgi?acc=GSE182127>) which consists of seven samples, among which are one control, five heme-hemopexin (10 nmol), four heme-albumin samples (0.30 nmol, 1.25 nmol, 5.0 nmol, 10 nmol respectively), and one sham samples. In the ST datasets, the representation of feature expression per spot is in the form of a sparse count matrix where the rows represent features, and the columns represent spots.

At first, we coerced the data using an in-built *Seurat* package in R-software and made a Seurat object from it. After generating the count matrix, we apply a normalization process using the "Relative Composition" (RC) method, which is built into the *Seurat* package. The RC normalization involves dividing the raw count data for each spot by the total number of reads in that spot, followed by multiplication with a scaling factor. We have used the default scaling factor that is 1e6, which is equivalent to multiplication by a million. This normalization is a crucial step to correct technical differences between cells that can result from differences in sample preparation, sequencing depth, and other experimental factors.

Next, we access the co-expression of some features of interest group 1 (*DDR1*, *C4B* and *GFAP*) and group 2 (*GAD1*, *GAD2*, *SLC17A6* and *SLC17A7*). To do so, we calculate the correlation coefficient between each of the 3 features in group 1 and 4 features in group 2. It therefore generates a 3 x 4 correlation matrix.

Subsequently, we generate a correlation plot utilizing the *image.plot* function from the *fields* package in R. The X-axis denotes the features of group 1, while the Y-axis represents those of group 2. The intensity of the deeper red color in the legend represents the degree of correlation between feature pairs, with higher intensity indicating stronger correlation.

Following this, we employ the *SpatialFeaturePlot* function within the Seurat package to visualize the expression of diverse features. This function overlays the expression levels of the chosen features on the tissue images at each spatial location.

**Supplementary figures**


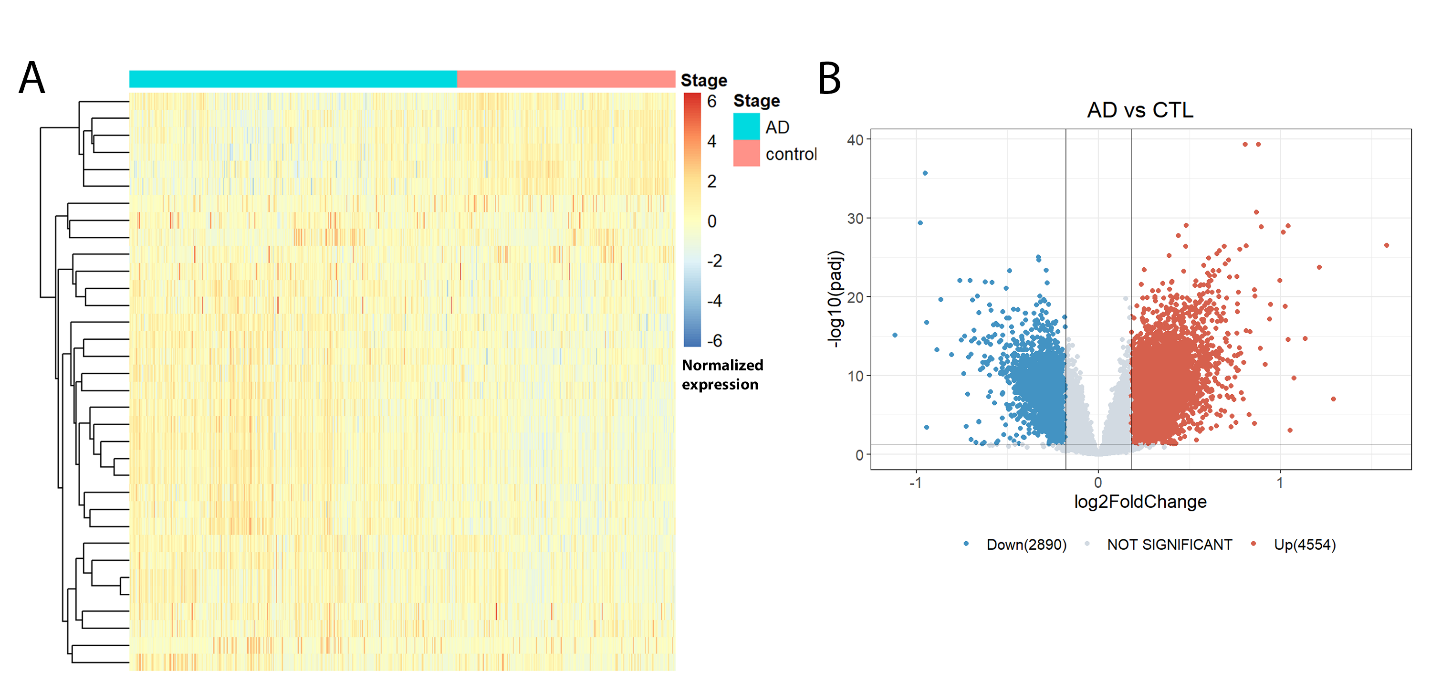


Fig. S1 Heatmap and volcano plot of up- and down- regulated genes in brains from AD samples vs control samples. (A) A hierarchical clustering heatmap displays gene expression changes in AD cohort between AD samples and control samples (FDR-adjusted *p*-value < 0.05 and absolute log2 FC > 0.80). The color scale represents normalized expression levels with performing *pheatmap*. (B) A volcano plot shows DEGs identified in AD cohort (FDR-adjusted *p*-value < 0.05 and absolute log2 FC > 0.18). In the heatmap and volcano plot, up-regulated genes are shown in red, while down-regulated genes are shown in blue.


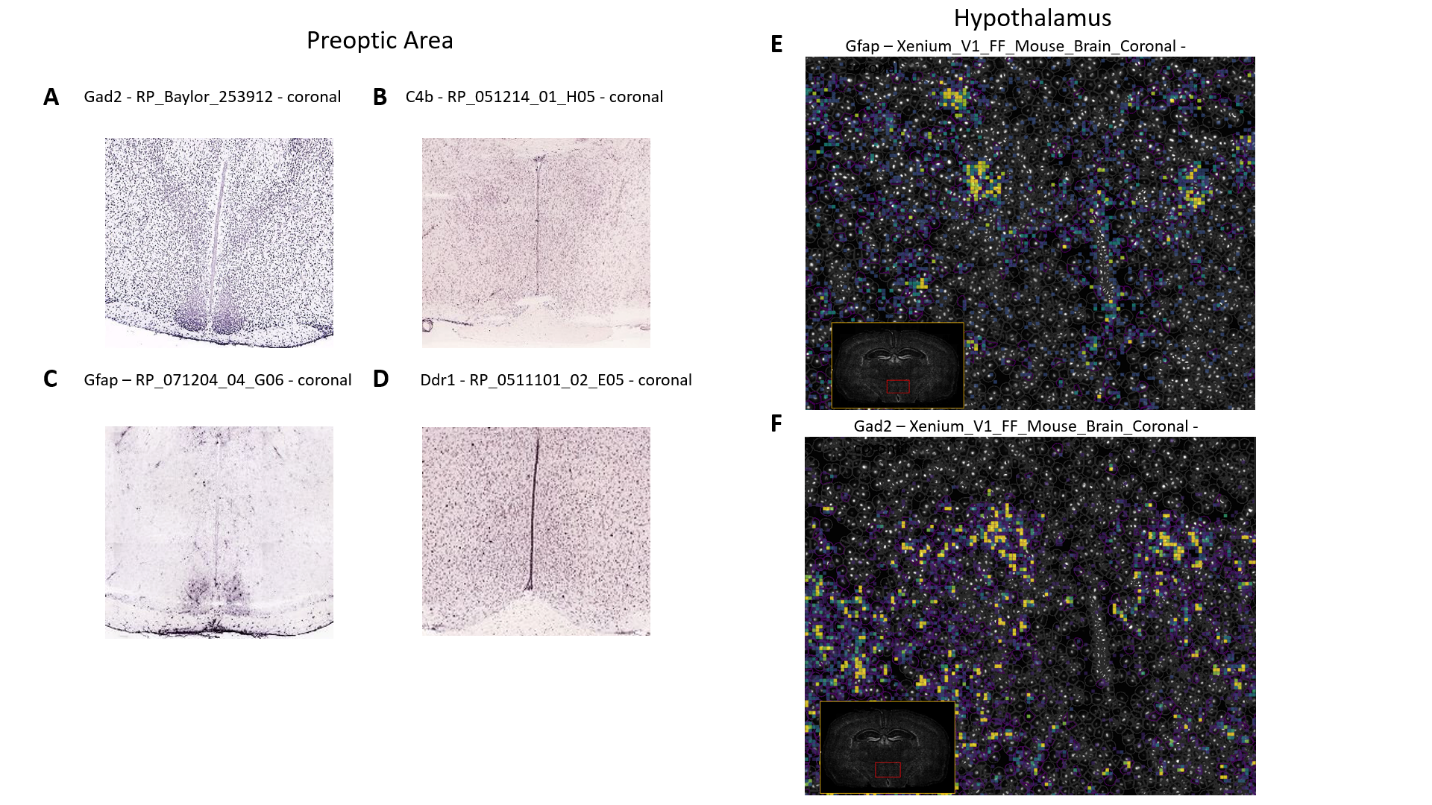


Fig. S2 Selected features expression in the mouse brain A-D) Immunohistochemistry (IHC) images of *Gad2, C4b, Gfap* and *Ddr1* protein expression in preoptic area of adult mouse brain. E-F) 10x Genomics Xenium images of *Gfap* and *Gad2* RNA expression in hypothalamus of mouse brain.


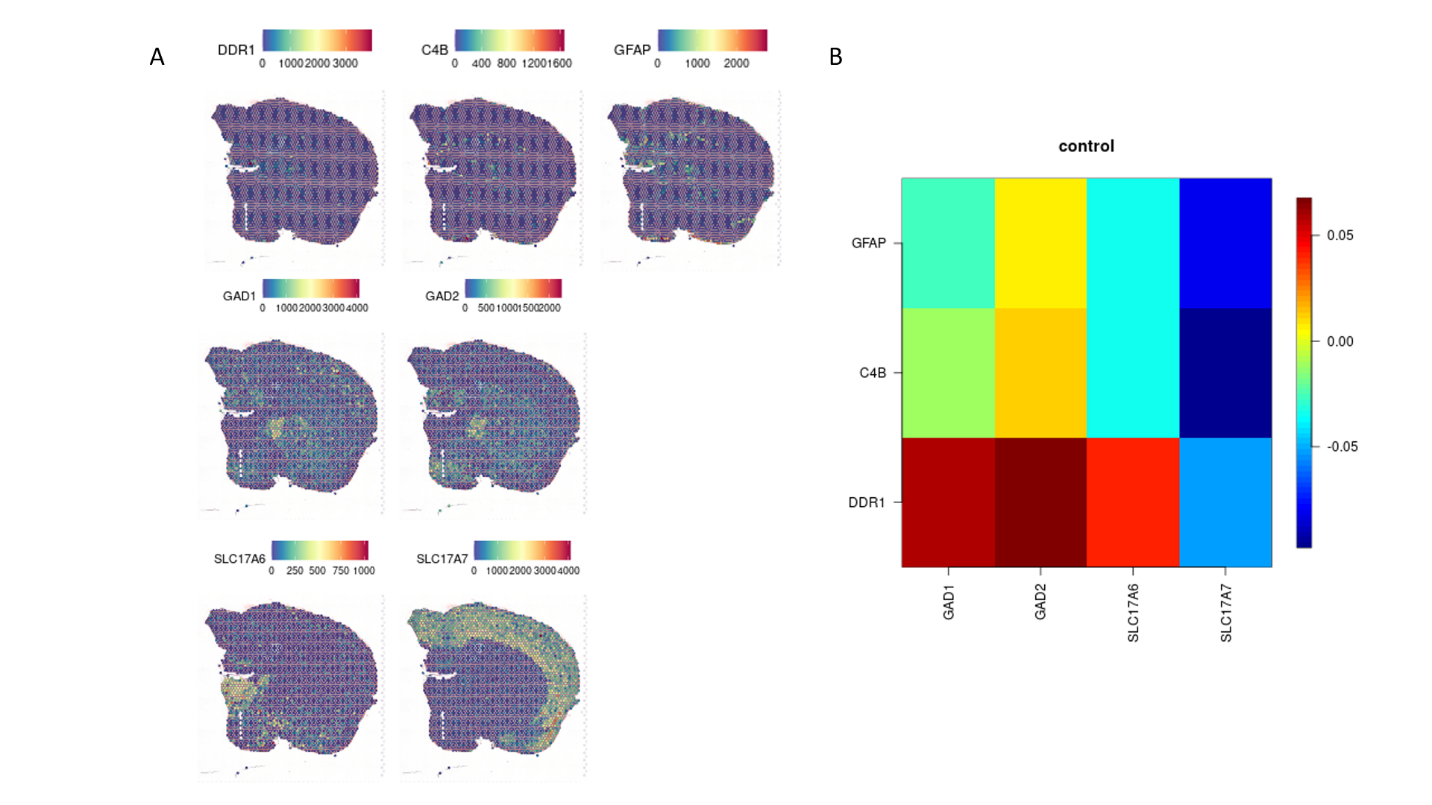


Fig. S3 Spatial gene expression pattern and the correlation of the selected features in control samples. A) 10x Genomics Visium images showing the feature expression score for the chosen features. B) Correlation plot between group 1 and group 2 among control samples.


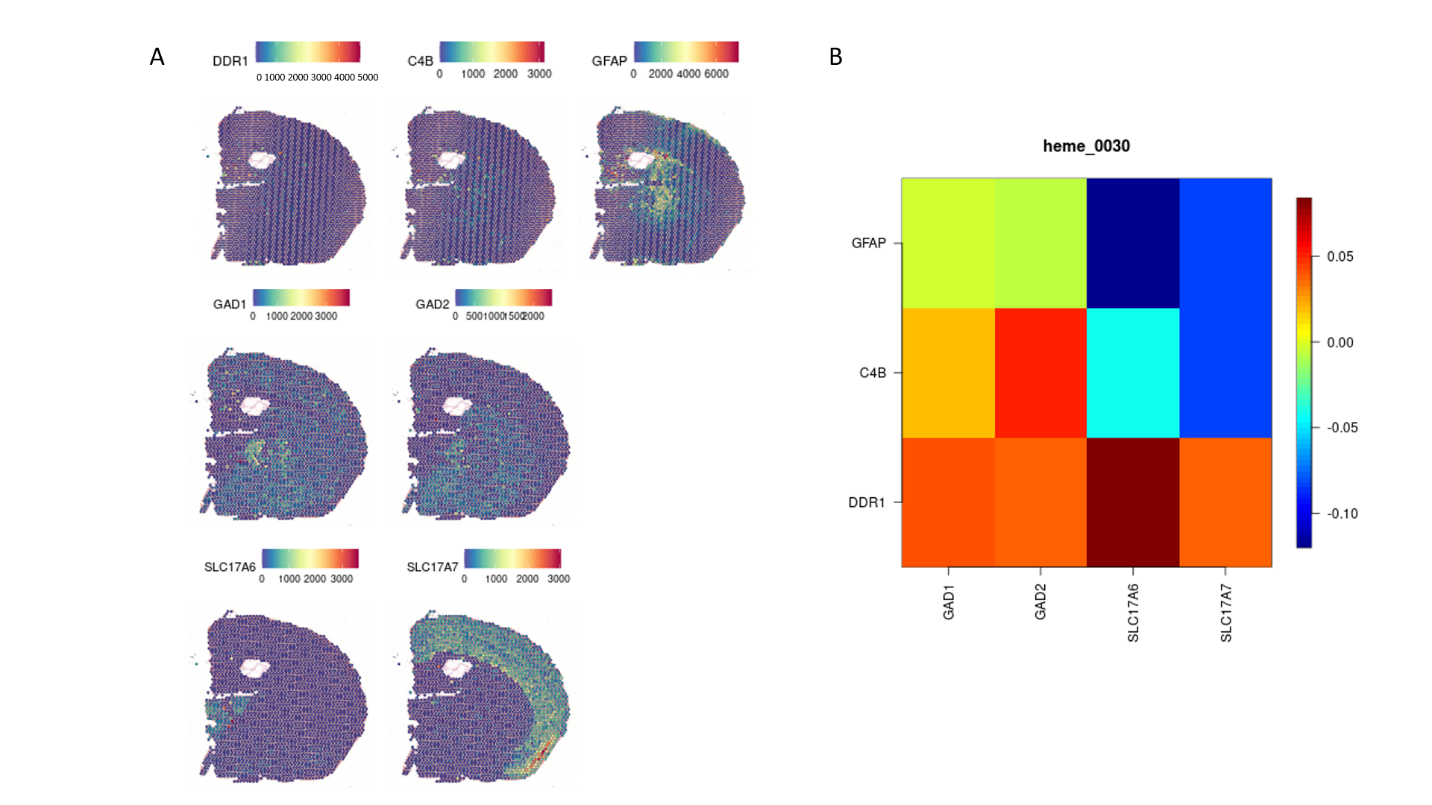


Fig. S4 Spatial gene expression pattern and the correlation of the selected features in 0.30 nmol heme-albumin treated samples. A) 10x Genomics Visium images showing the feature expression score for the chosen features. B) Correlation plot between group 1 and group 2 among 0.30 nmol heme-albumin treated samples.


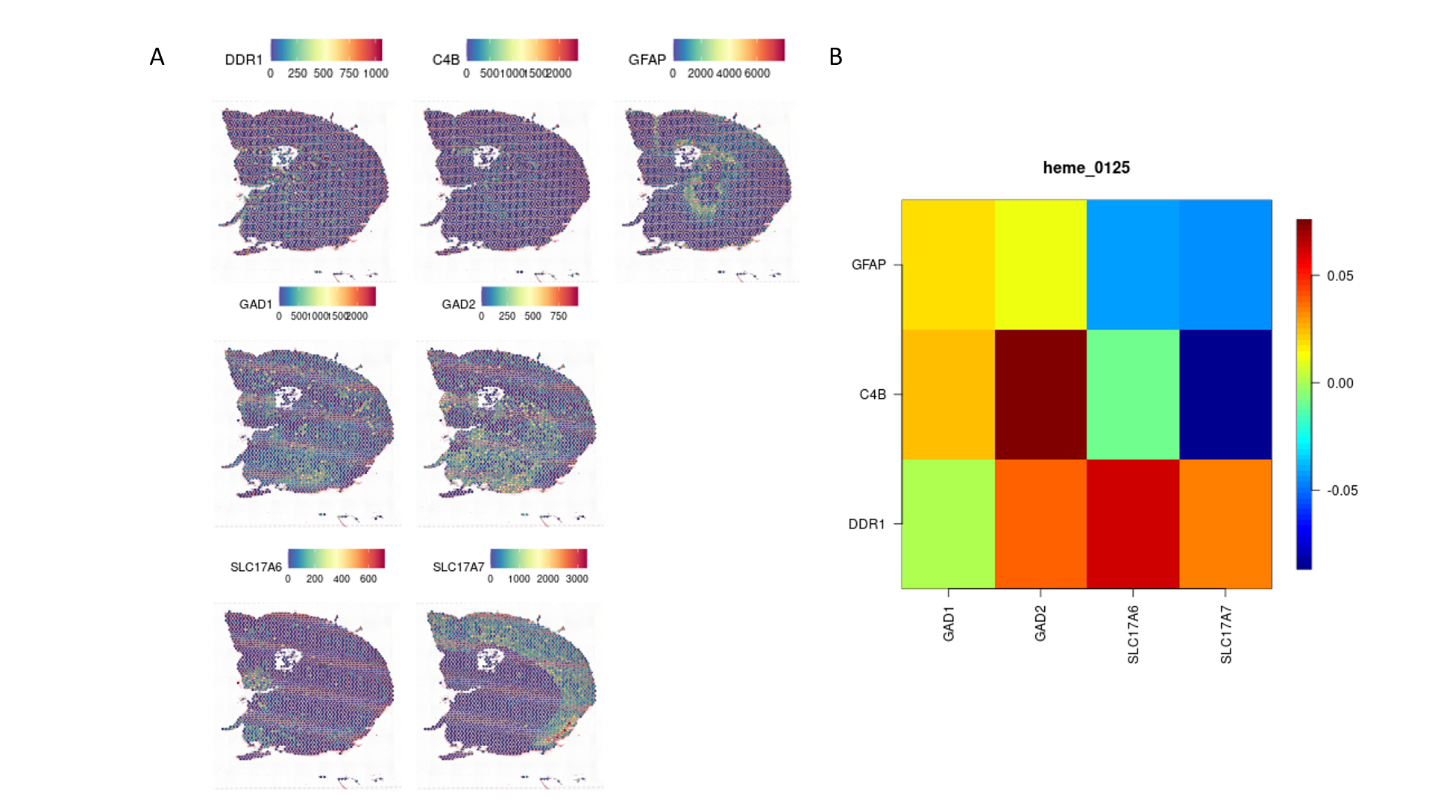
 Fig. S5 Spatial gene expression pattern and the correlation of the selected features in 1.25 nmol heme-albumin treated samples. A) 10x Genomics Visium images showing the feature expression score for the chosen features. B) Correlation plot between group 1 and group 2 among 1.25 nmol heme-albumin treated samples.


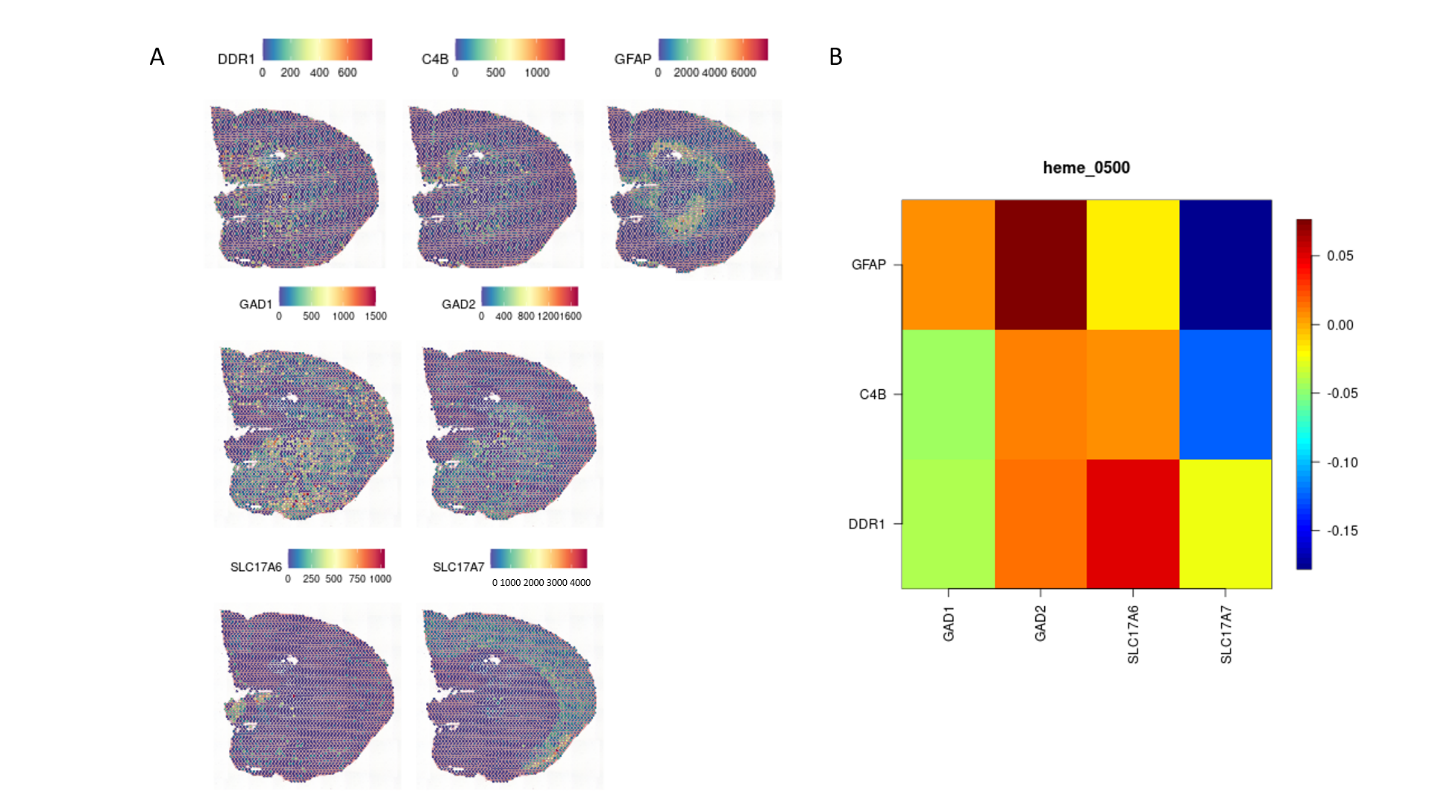


Fig. S6 Spatial gene expression pattern and the correlation of the selected features in 5.0 nmol heme-albumin treated samples. A) 10x Genomics Visium images showing the feature expression score for the chosen features. B) Correlation plot between group 1 and group 2 among 5.0 nmol heme-albumin treated samples.


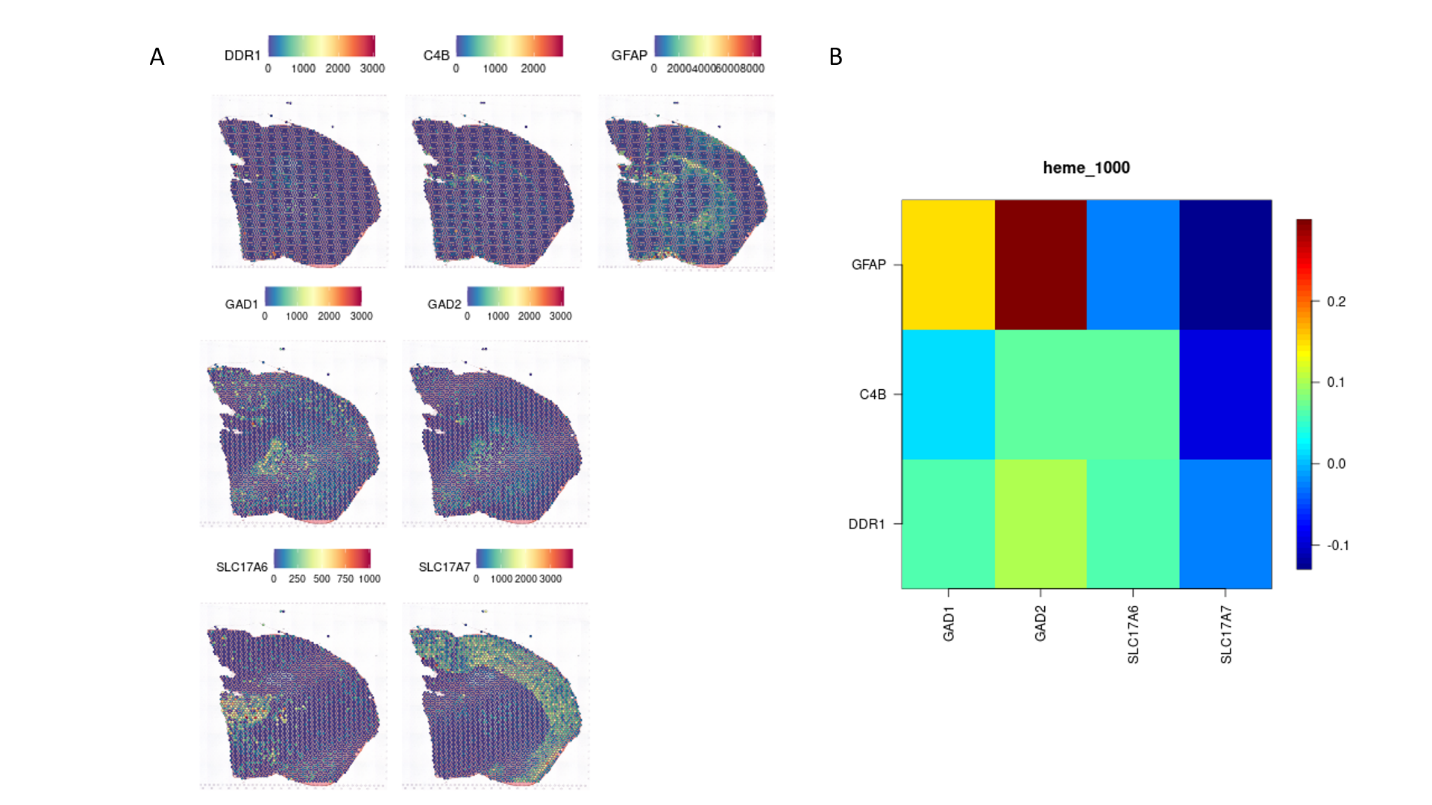


Fig. S7 Spatial gene expression pattern and the correlation of the selected features in 10.0 nmol heme-albumin treated samples. A) 10x Genomics Visium images showing the feature expression score for the chosen features. B) Correlation plot between group 1 and group 2 among 10.0 nmol heme-albumin treated samples.


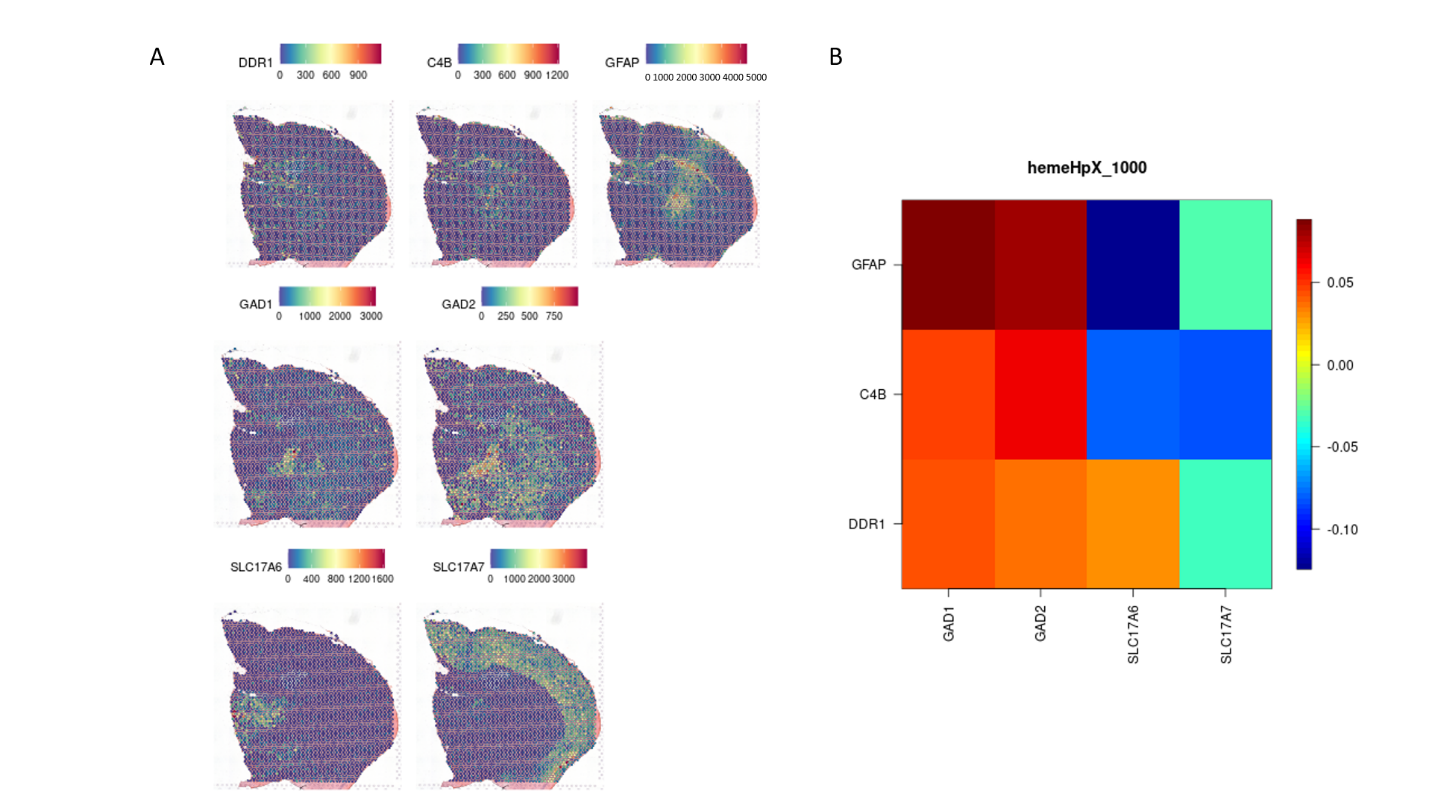


Fig. S8 Spatial gene expression pattern and the correlation of the selected features in 10 nmol heme-hemopexin treated samples. A) 10x Genomics Visium images showing the feature expression score for the chosen features. B) Correlation plot between group 1 and group 2 among 10 nmol heme-hemopexin treated samples.


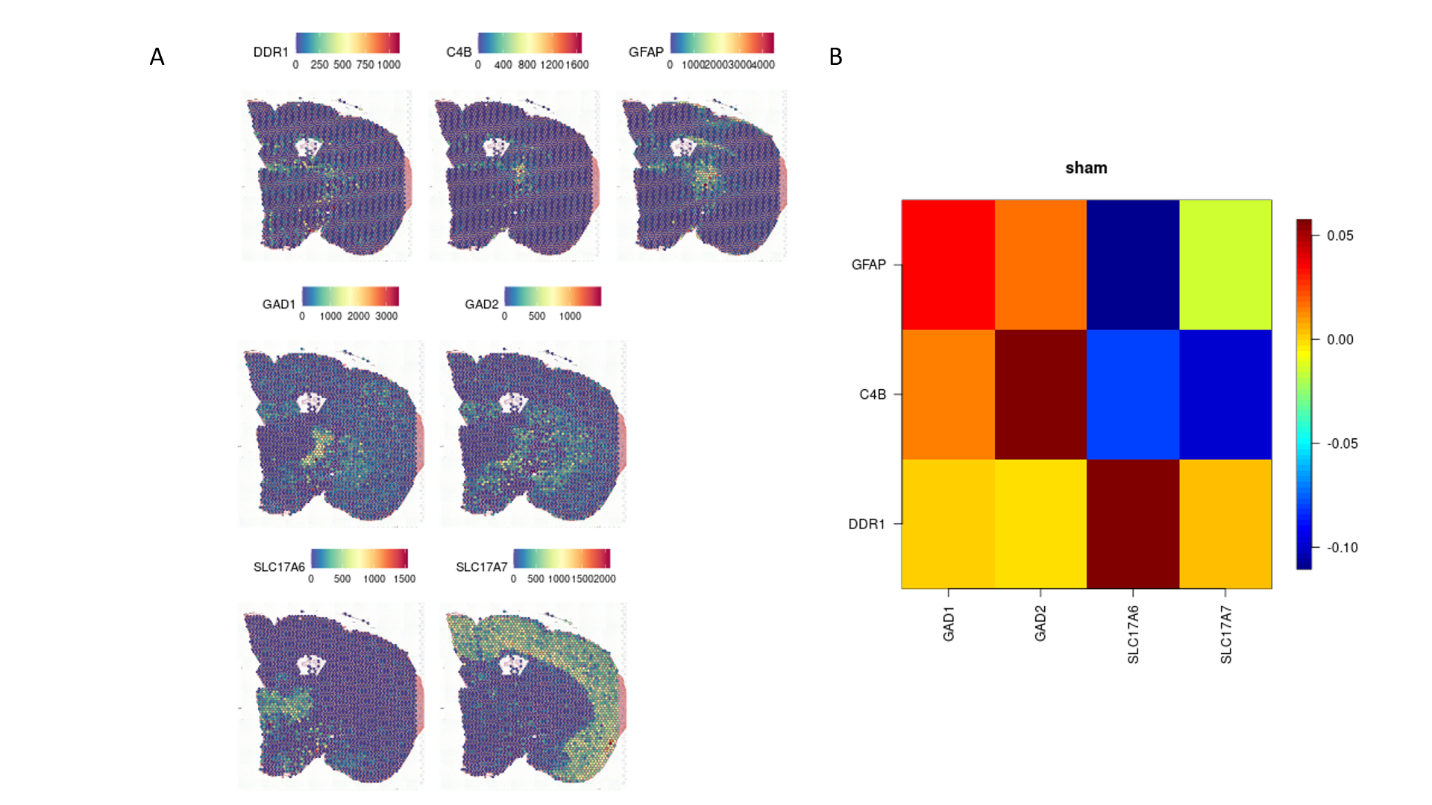


Fig. S9 Spatial gene expression pattern and the correlation of the selected features in sham samples. A) 10x Genomics Visium images showing the feature expression score for the chosen features. B) Correlation plot between group 1 and group 2 among sham samples.
